# *SAP30* Gene Is a Probable Regulator of Muscle Hypertrophy in Chickens

**DOI:** 10.3389/fgene.2021.709937

**Published:** 2021-09-27

**Authors:** Bruna Petry, Gabriel Costa Monteiro Moreira, Aline Gonçalves Lio Copola, Marcela Maria de Souza, Fernanda Cristina da Veiga, Erika Cristina Jorge, Jane de Oliveira Peixoto, Mônica Corrêa Ledur, James E. Koltes, Luiz Lehmann Coutinho

**Affiliations:** ^1^Animal Science Department, Luiz de Queiroz College of Agriculture (ESALQ), University of São Paulo, Piracicaba, Brazil; ^2^Department of Morphology, Institute of Biological Sciences, Federal University of Minas Gerais (UFMG), Belo Horizonte, Brazil; ^3^Animal Science Department, Iowa State University, Ames, IA, United States; ^4^Embrapa Suínos e Aves, Concórdia, Brazil

**Keywords:** hypertrophy, siRNA, knockdown, skeletal muscle, C2C12, muscle cell growth

## Abstract

Animals with muscle hypertrophy phenotype are targeted by the broiler industry to increase the meat production and the quality of the final product. Studies characterizing the molecular machinery involved with these processes, such as quantitative trait loci studies, have been carried out identifying several candidate genes related to this trait; however, validation studies of these candidate genes in cell culture is scarce. The aim of this study was to evaluate *SAP30* as a candidate gene for muscle development and to validate its function in cell culture *in vitro*. The *SAP30* gene was downregulated in C2C12 muscle cell culture using siRNA technology to evaluate its impact on morphometric traits and gene expression by RNA-seq analysis. Modulation of *SAP30* expression increased C2C12 myotube area, indicating a role in muscle hypertrophy. RNA-seq analysis identified several upregulated genes annotated in muscle development in treated cells (SAP30-knockdown), corroborating the role of *SAP30* gene in muscle development regulation. Here, we provide experimental evidence of the involvement of *SAP30* gene as a regulator of muscle cell hypertrophy.

## Introduction

Understanding the role of molecular gene networks in the regulation of the phenotype helps establish strategies to improve chicken production, increase product value, and reduce production costs. The intense genetic improvement that has occurred in recent years has increased poultry production, remodeling the industry over time (Saxena and Kolluri, 2018). Initially, chickens were selected for greater meat production by crossing lines previously selected for fast growth, carcass yield, and better feed conversion (Thiruvenkadan et al., 2011; Zuidhof et al., 2014). Despite the great advances in productive indexes, ongoing selection for growth rate, carcass, and prime cuts yields is correlated with pathophysiological changes in muscle tissue in modern lines, mainly the increase in breast myopathies (Soglia et al., 2019; Maharjan et al., 2020).

Advances in genomic technologies have led to discovery of single polymorphism nucleotide (SNP), development of SNP panels, and applications in animal (Dekkers, 2012). Aiming to identify genomic regions and putative candidate genes, several Genome-Wide Association Studies (GWAS) were conducted. Quantitative trait loci (QTL) have been identified in chickens for many traits of economic interest, including growth and body composition (Hu et al., 2019; Animal QTL Database, 2021), final body weight, gain, and feed efficiency (Hansen et al., 2005; Animal QTL Database, 2021). Our research group has characterized the genetic architecture for growth and body composition traits in an experimental population originated from a Brazilian broiler line (TT Reference Population). Candidate genes participating in the performance, organs, carcass traits (Venturini et al., 2015), and bone integrity (Fornari et al., 2014; Grupioni et al., 2017) were already discovered in this broiler population. Also, QTL associated with production traits, including carcass fat deposition (Moreira et al., 2018), feed efficiency, and body weight (Moreira et al., 2019). Using the same broiler population, a study searching for copy number variation regions (CNVR) was performed to find regions that can be important for the performance traits (Fernandes et al., 2021), and one of the CNVR was associated with breast yield (unpublished results).

Using the results from previous QTL (Trevisoli et al., 2021) and CNVR (unpublish results) analyses, a genomic region was associated with breast meat yield using information about TT Reference Population. Considering the possible importance of this region in the muscle architecture, we performed the data mining to search for genes that are annotated in this region and may present a role in muscle development. *SAP30* gene was found to be overlapping with a QTL and CNV region, both associated with musculature characteristics in the chicken population.

The *SAP30* gene is an important component of histone deacetylase complex in eukaryotic organisms (Zhang et al., 1998), and it has already been identified as a factor of transcriptional regulation in the gene expression in mouse skeletal muscle (Magnusson et al., 2005). This gene is related to the Sin3 proteins, acting as a binding protein important to repression and co-repression of gene transcription (Zhang and Iratni, 1997). *SAP30* gene has already been found to belong to a conserved region between chickens and mouse when comparing the chromosome organization (Viiri et al., 2009). The ancestral protein SAP30 was found to be conserved in many species of chloroplasts, fungi, plants, and animals, as well as an important conserved region called *GALNT-SAP30-HAND* according to the chromosome position between chicken and mouse (Viiri et al., 2009). Conservation across species is important because it allows studies to be carried out across different species that are expected to recapitulate the same effects. Thus, well-developed experimental tools such as cell lines in the mouse could be reasonably used to evaluate the function of a gene in the chicken or other livestock species.

To explore gene function, small interfering RNAs (siRNAs) are a versatile tool, particularly when used in cell lines that can be phenotyped across multiple experimental replicates in the lab. This technique has been widely used to inhibit mRNA transcription using a small sequence of RNA with mismatches in the target gene sequence under investigation (Bartel, 2004). In this way, siRNAs can silence a gene or simply reduce its expression (Morris et al., 2004), depending on the degree of sequence similarity to the target sequence. To understand the potential functional roles of mutations or genes in phenotype variation, functional experiments such as gene silencing or knockdown experiments must be conducted to eventually identify causal genes.

Given the economic importance of the genomic region containing QTL and CNVR for breast meat production in chickens and the fact that validation studies of gene function in cell culture are scarce, the objectives of this study were to select a candidate gene for muscle development and to validate its function in cell culture system (*in vitro*). Since the *SAP30* gene is well conserved across mouse and chicken, *in vitro* morphometric and gene expression analyses were performed on mouse C2C12 myoblast cells.

## Materials and Methods

### Population and Identification of Candidate Gene

The candidate gene selection was performed mining QTLs previously detected in a meat-type chicken population, called TT Reference Population. This is an experimental broiler population developed by the Embrapa Swine and Poultry National Research Center for genomics studies. The population was generated from the expansion of a paternal pure line (TT), and 20 males were mated with 92 females, generating approximately 1,500 chickens (Marchesi et al., 2018). In this population, QTLs were identified for breast muscle and yield. More specifically, one QTL on GGA4 (43 Mb) explained 0.61% of the genetic variance for breast muscle yield (Trevisoli et al., 2021), which overlapped with an inherited CNVR (GGA4: 43,406,643–44,413,344) associated with breast muscle weight and yield (unpublished data). These two previous studies show that this region may be participating in muscle development in chickens. Therefore, we used this information to search for the genes annotated in these regions.

Searches for deleterious and high-impact mutations were carried out, but no gene showed relevant mutations. Then, the next analysis for choosing the candidate gene was based on gene ontology terms for biological process, which could support the potential role of the candidate gene in muscle growth and/or development.

### C2C12 Cell Culture and Transfection

Cell culture and transfection were performed to characterize the *SAP30*-knockdown in the muscle cells. The C2C12 myoblasts (ATCC^®^ CRL 1772^TM^) were plated in a density of 2 × 10^4^ cell/well in growth medium [GM; Dulbecco’s modified Eagle’s medium (DMEM) with high glucose and L-glutamine (Gibco/ThermoFisher, Waltham, MA, United States)], supplemented with 10% fetal bovine serum (FBS; Gibco) and 1% penicillin/streptomycin/amphotericin B solution (Gibco/ThermoFisher, Waltham, MA, United States), in 24-well plates coated with 3% gelatin (Sigma-Aldrich). Transfection was performed when cells reached approximately 70% confluence, using a custom *SAP30* siRNA (ThermoFisher, Waltham, MA, United States) designed five target transcripts (NM_021788AF075136.1, AK010928.1, AK088745.1, BC132087.1, and BC132081.1) for the treated group (SAP30-knockdown cells). The Silencer negative control predesigned siRNA (ThermoFisher, Waltham, MA, United States) was used as the control group. The transfections were performed using Lipofectamine 3000 (ThermoFisher, Waltham, MA, United States), following the recommendations of the manufacturer, in triplicate for each group (control and treated). After 48 h at 37°C and 5% at CO_2_, the myogenic differentiation was induced by culturing cells at low serum conditions [DM: DMEM high glucose, supplemented with 2% horse serum (Gibco/ThermoFisher, Waltham, MA, United States) and 1% penicillin/streptomycin/amphotericin B (Gibco/ThermoFisher, Waltham, MA, United States) for 3 days.

### MTT Assay, Immunocytochemistry, Fusion Index, and Morphometric Analysis

Cell viability was checked based on mitochondrial activity using MTT [(3-(4,5-dimethylthiazol-2-yl)-2,5-diphenyltetrazolium bromide] assay. This method is capable of measuring cell viability, by means of colorimetric absorbance, based on mitochondrial cell activity. The amount of formazan blue crystals produced is proportional to the number of viable cells in the culture; thus, active cells produce more formazan crystals and thus more blue color than cells with low metabolic activity (Mosmann, 1983). This index was measured after 3 days in GM, in triplicate, and the absorbance was determined at 595 nm. Immunocytochemistry was performed to allow measuring myotube area only in differentiating cells. For that, the C2C12 cells (from treated and control groups) were fixed in 4% paraformaldehyde (PFA) for 15 min, permeabilized with 0.1% Tween 20, blocked with 1% BSA, and incubated with mouse anti-myosin antibody (MF20 monoclonal; Hybridoma Bank, diluted 1:200) at 4°C overnight. After washing, cells were incubated with secondary antibody peroxidase conjugated (EnVision^®^ Systems Dual Link, anti-mouse and anti-rabbit, Agilent Technologies, Santa Clara, CA, United States), signals were detected using DAB (Dako, Santa Clara, CA, United States), and the nuclei were counterstained with hematoxylin. All those analyses were performed in triplicate. Immunocytochemistry images were obtained using an inverted optical microscopy with capture system (BX41 Olympus and MoticAE31). The length and width of myotubes were obtained using ImageJ software. A stage micrometer was used for standardized analysis. Myotube area was determined by length × width, and the fusion index was determined by the ratio of the number of nuclei inside myotube cells over the total nucleus number 100, after 3 days in DM, following Martins et al. (2014) and Costa et al. (2021).

### RNA Isolation, Library Preparation, and Sequencing

Total RNA was isolated from each cell group (treated and control) in triplicate, using TRIzol (ThermoFisher, Waltham, MA, United States) reagent following the recommendations of the manufacturer. RNA integrity of the samples was determined using the Bionalyzer (Agilent Technologies, Santa Clara, CA, United States), and samples with RNA integrity number (RIN) score ≥ 7 were selected for library preparation. Libraries were prepared using the kit Illumina TruSeq Stranded mRNA Sample Prep LT Protocol (Illumina, San Diego, CA, United States), and sequencing was performed using the HiSeq SBS v4 kit to generate 100 bp (2 × 101) paired end sequence reads following the Illumina protocol. Samples were sequenced using HiSeq 2500 ultra-high-throughput system technology (Illumina, San Diego, CA, United States). The RNA sequencing was performed at the Genomic Center at ESALQ/USP (Piracicaba, São Paulo, Brazil).

### RNA Sequencing Bioinformatics: Quality Control and Read Mapping

Sequence quality control (QC) was evaluated using FastQC software^1^ (version 0.11.9). Library adaptors and low-quality sequences were trimmed away with TRIMgalore software^2^ (version 0.6.5), which removed the reads with Phred quality score lower than 20. Sequences reads were aligned against *Mus musculus* genome assembly (GRCh38.p6) using STAR software (Dobin et al., 2013; Supplementary Table 1). Gene counts were performed with FeatureCounts software (Liao et al., 2014, release 2.0.1), and the reads were normalized using TPM method with RSEM software (Li and Dewey, 2011). All subsequent statistical analysis were performed using R environment.

### Statistical Analysis and Differentially Expressed Genes

Differentially expressed (DE) genes were identified comparing control to *SAP30*-knockdown cells using DESeq2 software in R (Love et al., 2014). Reads were filtered as follows to exclude: (i) genes with no expression, i.e., gene annotations with zero reads; (ii) genes that may have aligned sequence due to mapping error, i.e., genes very lowly expressed with less than 1 read per sample on average; and (iii) genes rarely expressed across samples, i.e., genes with read counts that were not present in at least three (25% of all samples). To remove potentially batch effects, we used the Surrogate Variable Analysis (SVA) package (Leek et al., 2012). After these filters, a total number of 1,190 genes remained for DE analysis. The DE analysis included variables from the SVA analysis to remove batch effects as well as the treatment (control or SAP30-knockdown), which was the primary focus in identifying DE genes. Ontology enrichment analysis was performed using MetaCore software (MetaCore, 2021) to help interpret and summarize the cumulative biological process and pathways represented by DE genes.

## Results

### Identification of *SAP30* as a Candidate Gene for Muscle Development

A previous study from our group identified a QTL on GGA4 (43 Mb) associated with breast muscle yield (Trevisoli et al., 2021). In the same region, an inherited CNVR was identified (GGA4: 43,406,643–44,413,344) associated with breast meat weight and yield (unpublished results). These results provided us with two lines of evidence of the possible importance of this region for muscular development in chicken. We next searched for genes in this region and classified them by Biological Terms (Gene Ontology, GO terms). As shown in Table 1, *SAP30* was the only gene with a biological term related to skeletal muscle cell differentiation and, therefore, was the gene chosen as a candidate for the knockdown study.

**TABLE 1:** List of genes found within Quantitative Trait Loci (QTL) for breast yield in Genome-Wide Association Studies (GWAS) by Trevisoli et al. (2021) within the TT Reference Population at EMBRAPA.

Gene ID	Gene name	GO term name
ENSGALG00000010764	*FBXO8*	ARF guanyl-nucleotide exchange factor activity
ENSGALG00000020201	*CEP44*	Centrosome and spindle pole
ENSGALG00000010744	*GALNT7*	Transferase activity, membrane, golgi apparatus, and golgi membrane
ENSGALG00000010745	*HMGB2*	Regulation of stem cell proliferation
**ENSGALG00000036252**	***SAP30***	**skeletal muscle cell differentiation**
ENSGALG00000010755	*SCRG1*	Mesenchymal stem cell proliferation
ENSGALG00000030530	*HAND2*	Positive regulation of cardiac muscle hypertrophy
ENSGALG00000025263	*gga-mir-1776*	
ENSGALG00000032355		Golgi apparatus
ENSGALG00000030588		
ENSGALG00000033732		
ENSGALG00000037279		
ENSGALG00000032195		
ENSGALG00000033311		

*The list shows the genes found on chromosome 4 (43 Mb, 43,001,322–43,998,491) from the *Gallus gallus (Gallus_gallus*5.0, NCBI) genome build.*

### *SAP30* Gene Knockdown Promotes Muscle Cell Hypertrophy Phenotype

*SAP30* role during muscle cell proliferation and differentiation was investigated using the siRNA method. C2C12 cells were transfected with siRNA control or *SAP30* siRNA when cells reached 70% confluence. All analysis were performed after 3 days of cell culture in DM.

**FIGURE 1:**
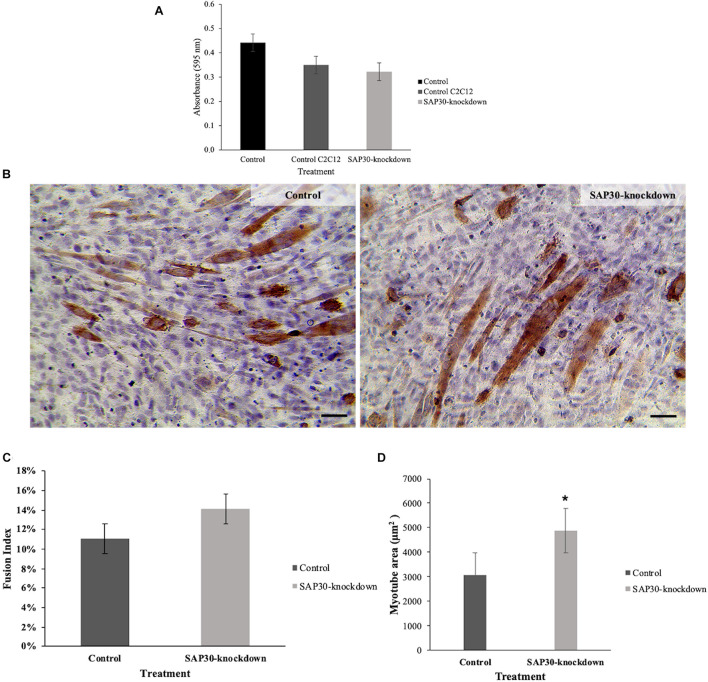
Cell viability index and morphological effects of C2C12 cells transfected with *SAP30* siRNA and control. **(A)** Graph representing the cell viability index determined by the MTT assay performed in C2C12 cells after 3 days of transfection with *SAP30* siRNA or siRNA control. **(B)** Representative image of MF20 immunocytochemistry result in C2C12 culture after 3 days of transfection with siRNA control and SAP30-knockdown. Scale bar = 100 μm. **(C)** Fusion index of MF20-positive cells transfected with siRNA control and *SAP30* siRNA after 3 days in DM. **(D)** Myotube area of C2C12 cells transfected with the siRNA control and SAP30-knockdown. Significant values *p < 0.05.

Before assessing the effects of *SAP30*-knockdown on cell morphology, we evaluated cell viability index via MTT assay after 3 days of cell culture to ensure that transfections were not toxic to the cells. We could observe no significant differences (*p* < 0.05) between control and *SAP30*-knockdown cells (Figure 1A), showing that the methodology used was not toxic for the cells and the morphological differences could be taken as exclusively due to the treatment.

Morphometric analysis showed an increase in myotube area of C2C12 cells that were transfected with siRNA for *SAP30* (SAP30-knockdown), compared to the control (Figure 1B). Cell fusion index was also assessed in order to determine if *SAP30*-knockdown could also be associated with skeletal muscle hyperplasia. However, our results indicated no significant difference (*p* < 0.05) between the control and *SAP30*-knockdown (Figure 1C). The myotube area was larger in the SAP30-knockdown group than in the control group (Figure 1D).

### Differentially Expressed Gene Analysis Revealed 1,190 Genes Being Differentially Expressed Between Control and Treated Groups

**FIGURE 2:**
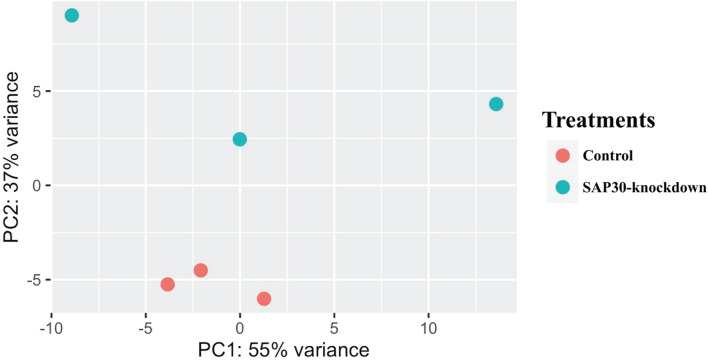
Principal Component Analysis (PCA) clearly showing a variation between genes expressed in the two groups (*SAP30*-knockdown and control) in C2C12 cells. The red dots are representing the triplicates from the control group, while the blue dots are triplicates from SAP30-knockdown group.

To investigate the mechanism by which *SAP30* gene was modulating myotube hypertrophy, we performed RNA sequencing and differential expression analysis. Initially, the differences in gene expression between the control and SAP30-knockdown groups were checked using a principal component analysis (PCA) plot. Figure 2 presents a PCA plot showing the triplicates from each group clustering together, and it is possible to observe a difference between both groups. The PC1 explains about 55% of the variance between the groups, and the PC2 explains about 37% of the variance.

**FIGURE 3:**
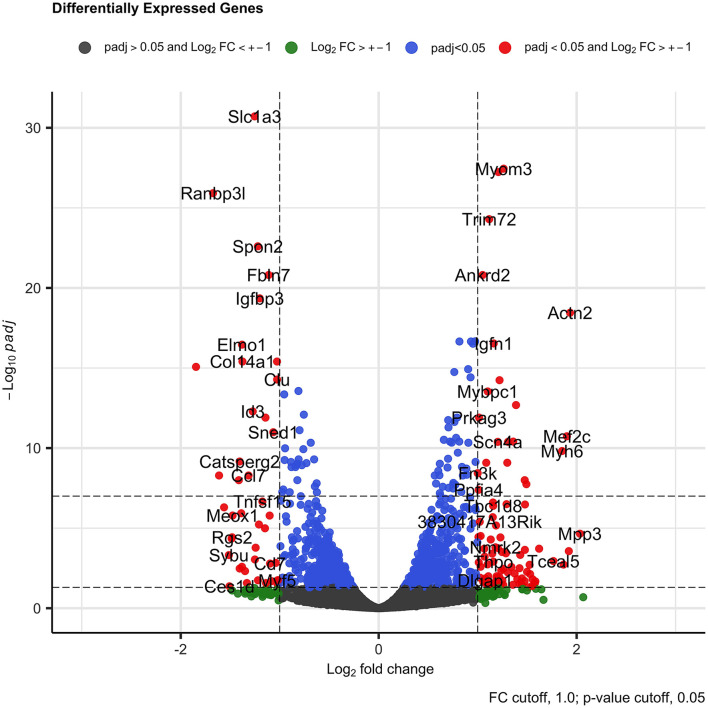
Volcano plot representing all genes with log2 fold change bigger than 1.0 and significant (*p* < 0.05) in the DE gene analysis in C2C12 cells after 3 days in differentiation medium. The red dots are the DE genes with log2 fold change higher than 1.0 in both sides (downregulated and upregulated) and with adjusted *p*-value < 0.05.

Differentially expressed gene analysis revealed a total of 1,190 (629 downregulated and 561 upregulated) genes were DE (*p*-adjusted < 0.05) when the control group was compared with the treated group (Supplementary Table 2). Several DE genes with log2 fold change higher than 1.0 for both sides (up- and downregulated) were identified (Figure 3). Among the downregulated genes, we found *Ranbp13* (Ran-binding protein 13), *Elmo1* (engulfment and cell motility 1), *Catsperg2* (cation channel sperm-associated protein subunit gamma 2), *Ccl7* (C-C motif chemokine ligand 7), Meox1 (mesenchyme homeobox 1), *Rgs2* (regulator of G protein signaling 2), *Sybu* (syntabulin), *NOS1* (nitric oxid synthase 1), *ABAT* (4-aminobutyrate aminotransferase), *Sectm1a* (secreted and transmembrane 1A), *Col14a1* (collagen type XIV alpha 1 chain), and *Vldlr* (very low-density lipoprotein receptor). We observed a small and not significant reduction in *SAP30* gene expression (log2 fold change = −0.17, *p*-adjusted = 0.53).

Among the upregulated genes, we found several genes related with muscle and actin process as *Actn2* (actin alpha 2), *Ablim3* (actin binding LIM protein family member 3), *Cbfa2t3* (CBFA2/RUNX1 partner transcriptional co-repressor 3), *Mef2c* (myocyte enhancer factor 2C), *Myh6* (myosin heavy chain 6), *Arhgef39* (Rho guanine nucleotide exchange factor 39), *Ablim2* (actin binding LIM protein family member 2), *Mpp3* (membrane palmitoylated protein 3), *Myh7* (myosin heavy chain 7), *Sdcbp2* (syndecan binding protein 2), *Clcnkb* (chloride voltage-gated channel kb), and *Igf2os* (insulin-like growth factor 2). These findings suggest the participation of the *SAP30* gene in the hypertrophy phenotype observed, since the reduction in the expression of the *SAP30* altered the expression of many muscle development markers.

### Enrichment Analysis Reveals Several Terms Related With Muscle Development

**FIGURE 4:**
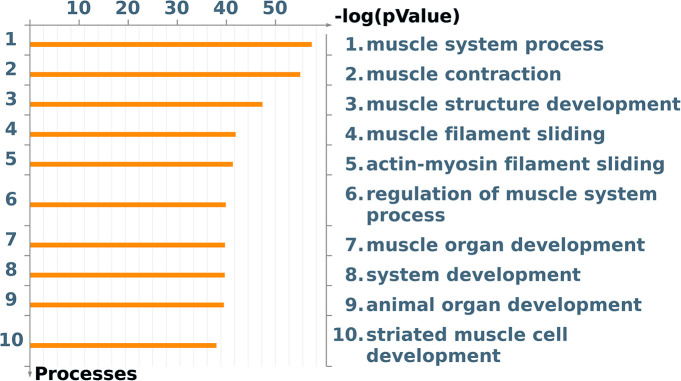
Gene Ontology terms showing the top 10 significant (*p* < 0.05) processes that the DE genes are related to. Image created by MetaCore software.

To identify the biological and cellular processes associated with *SAP30*, we performed an enrichment analysis. The top 10 GO terms were related with muscle system process, muscle contraction, muscle structure development, muscle filament sliding, actin-myosin filament sliding, regulation of muscle system process, muscle organ development, and striated muscle cell development (Figure 4).

**FIGURE 5:**
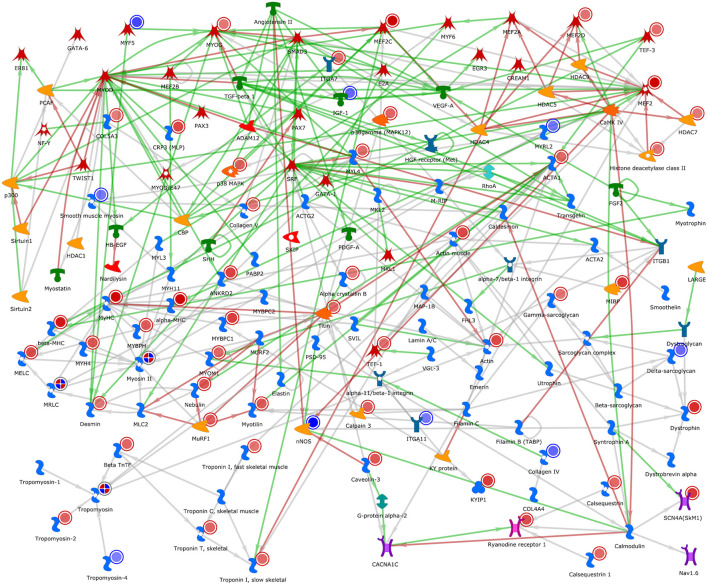
Developmental skeletal muscle network representing genes related with this process based on the DE genes analysis. The genes with red circles are genes that were upregulated after the DE analysis. Genes marked with blue circle were downregulated in our DE gene analysis. The red lines between the objects mean positive/activation effects; the green lines, negative/inhibition effects; and the gray lines, unspecified effects. Image created by MetaCore software.

Figure 5 shows the developmental skeletal muscle network presenting genes that were up- and downregulated in our study. Several genes associated with muscle skeletal development were upregulated (red circle), and among them were the transcription factors *MyoG*, *Mef2c*, *TEF-3*, and *MEF2D*, and the generic binding proteins *Actin*, *Tropomyosin-2*, *MyHC*, *Desmin*, *HYH4*, *Colagen 5*, *Dystrophin*, and *Troponin*. Among the downregulated genes (blue circle), we identified the transcription factor *MYF5*, a receptor ligand *IGF-1*, and the generic binding proteins *Colagen IV*, *Delta-sarcoglycan*, *MYRL2*, *Smooth muscle myosin*, and *Tropomyosin-4*, also a generic enzyme called *nNOS* and members of histone deacetylase complex (*HDCA1, HDCA4*, and *HDCA7*).

## Discussion

### *SAP30* as a Promising Candidate Gene Regulator of Muscle Hypertrophy

Our candidate gene, *SAP30*, is a Sin3A complex component. Some members of this family were already studied in C2C12 mouse myoblasts, revealing essential functions for Sin3 proteins in different stages of muscle development, where the Sin3A-null embryos for this protein do not survive the early stages of embryogenesis (van Oevelen et al., 2010). Studies with *SAP30* gene have shown its relationship with histone deacetylase process in eukaryotes that induces Sin3, the histone deacetylases HDAC1 and HDAC2 (Zhang et al., 1998), and acting as a transcription factor for *NETO2* gene in kidney cancer (Snezhkina et al., 2018). Besides that, *SAP30* has biological terms related with histone deacetylation, molecular function terms enriched for histone and protein deacetylase activity, cellular component for histone deacetylase complex, and histone deacetylase domain. In addition, one of those enriched terms is skeletal muscle cell differentiation, leading to a possible relation between this gene and muscle cell development.

To investigate the function of *SAP30* in muscle development, we performed gene knockdown in mouse myoblast cells (C2C12). We did not observe differences in myoblast proliferation (fusion index measurement), but morphometric analysis results revealed a potential role of the *SAP30* gene in the muscle cell hypertrophy. The cells that received the *SAP30* gene knockdown treatment developed a larger myotube area when compared with the control group. Myotubes are muscle differentiated cells, multinucleated, and formed by the fusion of the myoblasts to generate the myofibers and play a central role during the skeletal muscle formation and growth (Chal and Pourquié, 2017). The proliferation and differentiation of myoblasts were already associated with important economical traits in broiler chicken, including meat quality (Nihashi et al., 2019). A study measuring the muscle fiber diameters observed a positive correlation and participation of this phenotype in the breast weight and percentage in chickens (Koomkrong et al., 2015), also the fiber size, number, and composition are important traits related with meat quality (Lefaucheur, 2010; Wicks et al., 2019).

RNA-seq analyses revealed the expression of 19,489 genes, and among those, 1,190 genes were DE between the control and treated groups. Enrichment analysis of the DE genes revealed terms related to muscle system process, muscle structure development, muscle filament sliding, and actin-myosin filament sliding. The muscle system process term is responsible for muscle adaptation, contraction, and hypertrophy (Bult et al., 2019).

Muscle hypertrophy is an increase in the size of the myotube area that occurs due to the expression of myogenic markers capable of stimulating the production of muscle-specific proteins, as well as the activation and inhibition of metabolic pathways related to myogenic processes and tissue regeneration (Glass, 2005). In our study, we observed upregulation of many muscle contraction genes such as *Actn3*, *DMD*, *Myh3*, and *Myh7*, and some tropomyosin (*Tnnt2* and *Tnnt4)*. This observation agrees and helps explain the increase in myofiber diameter observed when *SAP30* was downregulated. Although the *SAP30* was not DE in the analysis, our results clearly demonstrates that cells treated with siRNA for *SAP30* during the proliferating phase results in upregulation of muscle contraction genes and hypertrophy.

To further explore how *SAP30* could modulate myoblast hypertrophy, it is important to investigate the expression of transcription factors associated with muscle cell determination and differentiation. Transcription factors such as *Myf5, MyoD, Mef2c, MRF4, Myf6*, and *Myogenin* are involved in the myogenic program (Zammit, 2017). The myocyte enhancer factor 2c (*Mef2c*) is a member of the Mef2 family of transcription factors and cooperates with MyoD family of basic–loop–helix (bHLH) transcription factors to play a role during skeletal muscle development (Molkentin et al., 1995; Potthoff et al., 2007). *Mef2c* knockout mouse specifically in skeletal muscle abnormalities in the myofiber perinatally structure and the adults that survived had some alterations in the skeletal myofiber types (Potthoff et al., 2007). In our work, we observed upregulation of *MefF2c* transcripts in siRNA-SAP30-treated cells.

In chicken and mouse embryos, activation of the myogenic factor *Myf5* is an important signal for skeletal muscle development in the somites (Ott et al., 1991; Pownall and Emerson, 1992). Other studies reported that downregulation of *Myf5*, induced by *MyoD* gene, results in an increase in expression of myogenin, promoting cell differentiation (Deato et al., 2008; Liu et al., 2012; Hernández-Hernández et al., 2017). These results corroborate with our study where myogenin was upregulated and the *Myf5* was downregulated in *SAP30*-knockdown C2C12 cells. Also, when we are representing the skeletal muscle development pathway, it is possible to observe a positive *Myf5* gene relation with *Desmin* and *Myogenin* (*MyoG*).

During muscle development, *MyoG* gene is expressed after *Myf5* (Francetic and Li, 2011). In studies with mice MyoG-null mice, it was observed that these animals had severe deficiency in the formation of skeletal muscle, where the formation of myoblasts occurred, but the myoblast fusion to form myotubes does not occur, leading these animals to an early death (Venuti et al., 1995). *Desmin* is a gene responsible to intermediate filament that integrates the Z-disk, sarcomers, nuclear membrane, and sarcolemma, being a muscle-specific protein (Brodehl et al., 2018). A study with a Desmin knockout mice shows a rigidity of the musculature, with a large amount of collagen and thes expression of genes related to the renewal of the extracellular matrix and inflammatory proteins, suggesting that the imbalance in the expression of this gene can cause cellular injuries and fibrosis (Meyer and Lieber, 2012). Missense mutations in this gene were also associated with cardioskeletal myopathy (Goldfarb et al., 1998). These are important evidence of the *Myogenin* and *Desmin* participation in the phenotype, which corroborates with our findings where both genes were upregulated when there is cellular muscle hypertrophy.

Not only *Myogenin* and *Desmin* but also many other genes already described to be related to different stages of skeletal muscle development, such as *MyHC* (Agarwal et al., 2020), *Dystrophin* (Stone et al., 2005), *Actin* (Bertola et al., 2008), *Mef2* (Taylor and Hughes, 2017), different tropomyosin (Gunning et al., 2008), and some collagen proteins (Moore et al., 2005), were found to be upregulated when the *SAP30* was knocked down in skeletal muscle cells.

These findings suggested *SAP30* as a possible regulator of muscle growth and development, since its knocking down induces genes and transcription factors responsible for increasing myotube differentiation and the formation of muscle myofibers. Possibly, there may be an explanation for the fact that this gene is close to a CNV region; however, further studies of overexpression and looking for functional mutations must be carried out to understand the role of the *SAP30* gene in the regulation of the muscle hypertrophy.

## Conclusion

For the first time, we showed morphometric and molecular evidence of the possible participation of *SAP30* gene in the muscle hypertrophy regulation. Even though there is a slight change in its expression, many genes and muscle regulators had their expression enhanced while the myotubes were also increased. Therefore, we have shown that the *SAP30* gene is a candidate for muscle development and cell hypertrophy regulation.

## Data Availability Statement

The datasets presented in this study can be found in online repositories. The names of the repository/repositories and accession number(s) can be found below: European Nucleotide Archive, accession no: PRJEB45219.

## Ethics Statement

The animal study was reviewed and approved by Embrapa Swine and Poultry Ethics Committee on Animal Utilization (CEUA), number 010/2010, in Concordia, Santa Catarina State, Brazil. The CEUA is in agreement with the rules of National Council of Animal Experimentation Control (CONCEA) to ensure compliance with international guidelines for animal welfare.

## Author Contributions

BP, GM, EJ, ML, JP, and LC worked on the organization and idea of the project. ML, JP, EJ, JK, and LC provided the experimental environment, laboratories, and support for data analysis. BP, AC, and EJ worked on the experimental part of cell culture. BP, MS, and GM performed the data analysis. BP drafted the manuscript. BP, GM, MS, AC, FV, EJ, JK, JP, ML, and LC collaborated with the interpretation of results, discussion, and wrote the manuscript. All the authors have read and approved the final manuscript.

## Conflict of Interest

The authors declare that the research was conducted in the absence of any commercial or financial relationships that could be construed as a potential conflict of interest.

## Publisher’s Note

All claims expressed in this article are solely those of the authors and do not necessarily represent those of their affiliated organizations, or those of the publisher, the editors and the reviewers. Any product that may be evaluated in this article, or claim that may be made by its manufacturer, is not guaranteed or endorsed by the publisher.

## Footnotes

1
https://www.bioinformatics.babraham.ac.uk/projects/fastqc/

2
https://www.bioinformatics.babraham.ac.uk/projects/trim_galore/

## Supplementary Material

Supplementary Table 1 Summary of total reads before and after filtering, percentage of uniquely mapped reads and unmapped reads alignedagainst Mus musculus genome assembly (GRCh38.p6) and groups (treated and control).

Supplementary Table 2 List with 1,190 DE genes.
